# Pediatric complex Chiari I Malformation—how complex is it?

**DOI:** 10.1007/s00381-026-07325-6

**Published:** 2026-05-20

**Authors:** Amparo Saenz, Honglin Zhu, Jasneet Dhaliwal, Sniya Sudhakar, Kshitij Mankad, Dominic Thompson, M. Zubair Tahir

**Affiliations:** 1 Pediatric Neurosurgery Department, Great Ormond Street Hospital, London, UK; 2 Pediatric Neuroradiology Department, Great Ormond Street Hospital, London, UK

**Keywords:** Pediatric neurosurgery, Chiari malformation, Foramen magnum decompression, Spine

## Abstract

**Background:**

Pediatric complex Chiari I Malformation (CCM), defined by Chiari I with additional craniovertebral junction anomalies, has been associated with higher failure rates following foramen magnum decompression (FMD) alone, leading some to advocate occipitocervical fixation (OCF) or ventral decompression. This study evaluates a single-institution experience with isolated FMD in CCM.

**Methods:**

Patients meeting CCM criteria were identified from a prospective surgical database from March 2020 to December 2023. Patients with incomplete imaging or < 6-month follow-up were excluded. Pre- and postoperative imaging assessed tonsillar descent, brainstem crowding, CSF flow, and syringomyelia. Clinical outcome was recorded.

**Results:**

Sixty patients met the inclusion criteria for CCM, but nineteen patients were excluded. Forty-one CCM patients underwent FMD. Headache prevalence decreased from 73.2% preoperatively to 7.3% at last follow-up (*p* < 0.001). One patient (2.4%) required reoperation for inadequate decompression. Median tonsillar descent improved from 15.0 to 0.0 mm (*p* < 0.001), and obex ectopia from 7.0 to 0.0 mm (*p* < 0.001). Anterior and posterior CSF effacement significantly improved. Craniocervical angle increased from 122.0 to 130.0° (*p* = 0.007), and pb–C2 distance decreased from 7.0 to 5.0 mm (*p* < 0.001). No patient required OCF or ventral decompression during a mean 2.1-year follow-up.

**Conclusion:**

Isolated FMD provides effective clinical and radiological improvement in most pediatric CCM patients. Routine addition of OCF or ventral decompression may be unnecessary, preserving cervical mobility while minimizing morbidity.

## Introduction

Chiari I Malformation (CM), characterized by the caudal displacement of cerebellar tonsils below the foramen magnum, is a well-recognized neurological condition often associated with cerebrospinal fluid (CSF) flow disruption and a spectrum of debilitating symptoms [1, 2]. Complex Chiari I Malformation (CCM) [3–6] refers to a subgroup of CM patients who have additional craniovertebral junction (CVJ) anomalies comprising obex ectopia, medullary kinking, retroflexed odontoid, or basilar invagination, which individually or in combination contribute to ventral brainstem compression [7]. It has been suggested that clinical and radiological outcomes are less favorable in these patients.

While foramen magnum decompression (FMD) is the cornerstone of treatment for classical Chiari I [7–10], the presence of associated CVJ anomalies in CCM has led some to advocate for routine occipitocervical fixation (OCF) in addition to FMD [3, 4, 11]. Proponents of OCF argue that it directly addresses underlying instability and anterior compression, which FMD alone may not fully resolve. Conversely, the potential for increased morbidity, loss of cervical spine mobility, and limited long-term evidence supporting routine OCF in this population raises concerns.

This study was aimed at evaluating the safety and efficacy of isolated foramen magnum decompression (FMD) in a cohort of pediatric patients with CCM. Through analysis of clinical outcomes and radiological changes following isolated FMD, we sought to determine whether this less invasive approach can effectively alleviate symptoms, resolve associated pathologies like syringomyelia, and achieve durable neurological improvement without the routine necessity of occipitocervical fixation.

## Methods

This retrospective observational study was conducted at the Paediatric Neurosurgery Department of Great Ormond Street Hospital (GOSH) in London. CCM was defined as Chiari I Malformation associated with at least one of the following craniovertebral junction (CVJ) anomalies: basilar invagination, retroflexed odontoid, craniocervical deformity (CXA), medullary kinking, or atlantoaxial instability.

The study included all patients who underwent surgical treatment for CCM between March 2020 and December 2023. Patients were identified from a departmental prospectively populated operative database. Clinical and radiological data were collected from the electronic medical records and assessment of preoperative and postoperative imaging studies.

Radiological evaluation was performed using preoperative and postoperative magnetic resonance (MR) and computed tomography (CT) scans**.** Cerebellar tonsillar ectopia, measured from the tip of the tonsils to the McRae line, was considered pathologic if the descent exceeded 5 mm [2, 12]. Obex ectopia was defined as any descent of the obex below the McRae line. Anterior and posterior cerebrospinal fluid (CSF) spaces at the foramen magnum were measured, with effacement defined as < 2 mm [7].

Retroflexion of the odontoid process was assessed using the pb–C2 line [5], calculated as the perpendicular distance from the ventral dura to a line drawn from the basion to the posterior inferior corner of C2. A value > 9 mm was considered indicative of a retroflexed odontoid [5, 13]. Craniocervical deformity was assessed using the clival-cervical angle (CXA), formed between the clivus and the anterior cervical spine, with angles < 125° considered abnormal [4, 14, 15].

Additional craniovertebral junction anomalies included basilar invagination (BI), defined as an odontoid tip projecting > 5 mm above the McGregor line [16, 17]. The basion–dens interval (BDI), measured as the distance between the basion and the tip of the odontoid process, was considered abnormal if > 10 mm. The basion–axial interval (BAI), measured as the distance between the basion and a line drawn perpendicular to the posterior cortex of the C2 vertebral body through the odontoid tip, was considered abnormal if > 12 mm [18, 19].

The skull base angle was also measured, defined as the angle between a line extending along the anterior cranial fossa to the tip of the dorsum sellae and a line along the posterior margin of the clivus. Angles > 120° were classified as platybasia, < 110° as basilar kyphosis, and 110–120° as normal [20].

Instability was assessed through systematic morphometric analysis of all standard craniocervical parameters on preoperative MRI and CT in all patients. Flexion–extension plain radiographs were obtained selectively in patients with clinical suspicion of instability or borderline morphometric values. In the remaining patients, the absence of instability-related symptoms and the normal BDI, BAI, and atlantodens interval on static imaging were used to exclude significant instability, in keeping with CNS guideline recommendations [21].

The presence of syringomyelia (documenting the maximum syrinx diameter and number of vertebral levels involved) and syringobulbia was also documented.

All measurements were made on mid-sagittal T1- and T2-weighted MRI sequences, and when available, on sagittal CT sequences, except for CSF effacement and syrinx diameter, which required axial T2-weighted MRI sequences. To locate the obex, the method of H. E. Moore and K. R. Moore [7] was used, tracing inferiorly along the floor of the fourth ventricle to the superior margin of the dorsal medullary contour change on a midline sagittal T2 MR image. For both tonsillar and obex descent, the distance below the McRae line was measured, with any value at or above this line recorded as 0.

Postoperative radiological assessment was conducted on follow-up MRI and, when available, CT scans. The postoperative images were performed at least six months after surgery. Changes in morphometric parameters were recorded and compared to preoperative values to assess decompression efficacy and brainstem–spinal cord realignment.

In addition to radiological analysis, preoperative and postoperative symptomatology was recorded for each patient, including the presence or absence of headache, neck pain, arm pain, back pain, weakness, and sensory disturbances. Clinical follow-up was completed at the last recorded follow-up. Reoperation for any reason and surgical complication rates were also documented.

The study protocol was approved by the institutional Research and Ethics Committee and was done in accordance with the ethical standards of the institutional and national research committee and with the 1964 Helsinki Declaration and its later amendments or comparable ethical standards.

Statistical analysis was performed using Stata version 16.1 (StataCorp, College Station, TX), and graphs were done with R Studio. Normality of continuous variables was assessed using the Shapiro–Wilk test. Parametric or non-parametric tests were selected accordingly. Categorical variables were presented as absolute frequencies and percentages, and continuous variables as mean and standard deviation (SD) or median and interquartile range (IQR), depending on their distribution. Paired *t*-tests or Wilcoxon signed-rank tests were used to compare continuous radiological measures before and after surgery. McNemar’s test was applied to evaluate paired binary outcomes, such as the presence of symptoms. A *p*-value of less than 0.05 was considered statistically significant. For multiple comparisons of radiological parameters, *p*-values were adjusted using the false discovery rate (FDR) method, with the same threshold applied to the adjusted values.

## Results

Between March 2020 and December 2023, patients underwent surgery for Chiari I Malformation. Of these 60 patients met the inclusion criteria for CCM. Nineteen patients were excluded due to insufficient follow-up or incomplete data. None of the patients excluded required secondary surgery. A total of 41 patients were analyzed for this study.

**Fig. 1:**
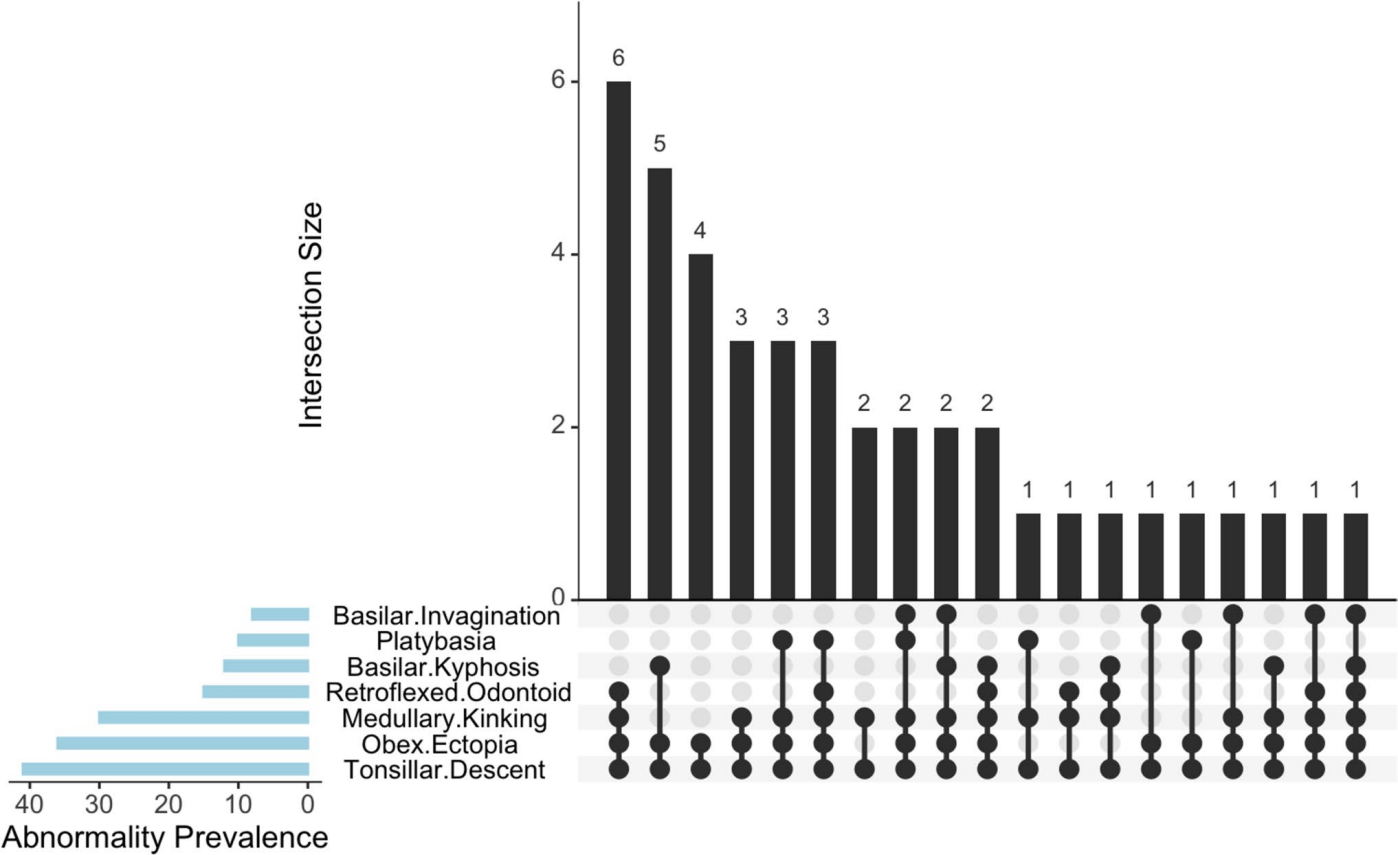
Overlap and prevalence of pre-operative craniovertebral junction abnormalities. An UpSet plot illustrating the prevalence of individual structural abnormalities (left bar chart) and their intersections (top bar chart) among the pediatric Complex Chiari I Malformation cohort. Dots indicate the combination of abnormalities present in each intersection

The distribution and overlap of the complex features are depicted in Fig. 1, illustrating the most common combinations. At presentation, the median tonsillar descent was 15 mm (IQR 11–21), and the median obex ectopia, measured in patients with confirmed ectopia (*n* = 36), was 8 mm (IQR 3.5–12). Anterior CSF space effacement was present in 80.5% (*n* = 33), and posterior effacement in 90.2% (*n* = 37) of cases. Craniocervical deformity, measured by the CXA angle, was observed in 73.1% (*n* = 30) of cases. In those affected, the mean CXA angle was 117.1° (SD ± 9.2), with a median of 122° (IQR 116–128) across affected individuals. Retroflexion of the odontoid was present in 36.6% (*n* = 15) of patients, with a mean pb–C2 distance of 10.2 mm (SD ± 0.8) in this subgroup. The mean pb–C2 distance in the entire cohort was 7.3 mm (SD ± 2.6). BI was present in 19.5% (*n* = 8) of cases. In these patients, the median invagination distance was 6.5 mm (IQR 6–7.75). The overall median invagination distance was 0.0 mm (IQR 0–4.3). One patient (2.4%) had a median BDI of 11 mm (IQR 11–11), and two patients (4.8%) had a median BAI of 13 mm (IQR 13–13). The overall mean BDI for the cohort was 5.7 mm (SD ± 2.1), while the mean BAI was 7.0 mm (SD ± 2.8). In terms of skull base morphology, 24.4% (*n* = 10) of patients were classified as having platybasia, 29.3% (*n* = 12) had basilar kyphosis, and 46.3% (*n* = 19) had a normal skull base angle. The mean skull base angle was 113.9° (SD ± 9.1) for the entire cohort. Syringobulbia was identified in 19.5% (*n* = 8) of patients, while syringomyelia was present in 75.6% (*n* = 31) of patients. The median transverse diameter of the syrinx was 7.1 mm (IQR 4.5–9.7), while the median length was 14 spine levels (IQR 6–17). Eleven patients (26.8%) had flexion-extension XR preoperatively, and none showed instability.

**Fig. 2:**
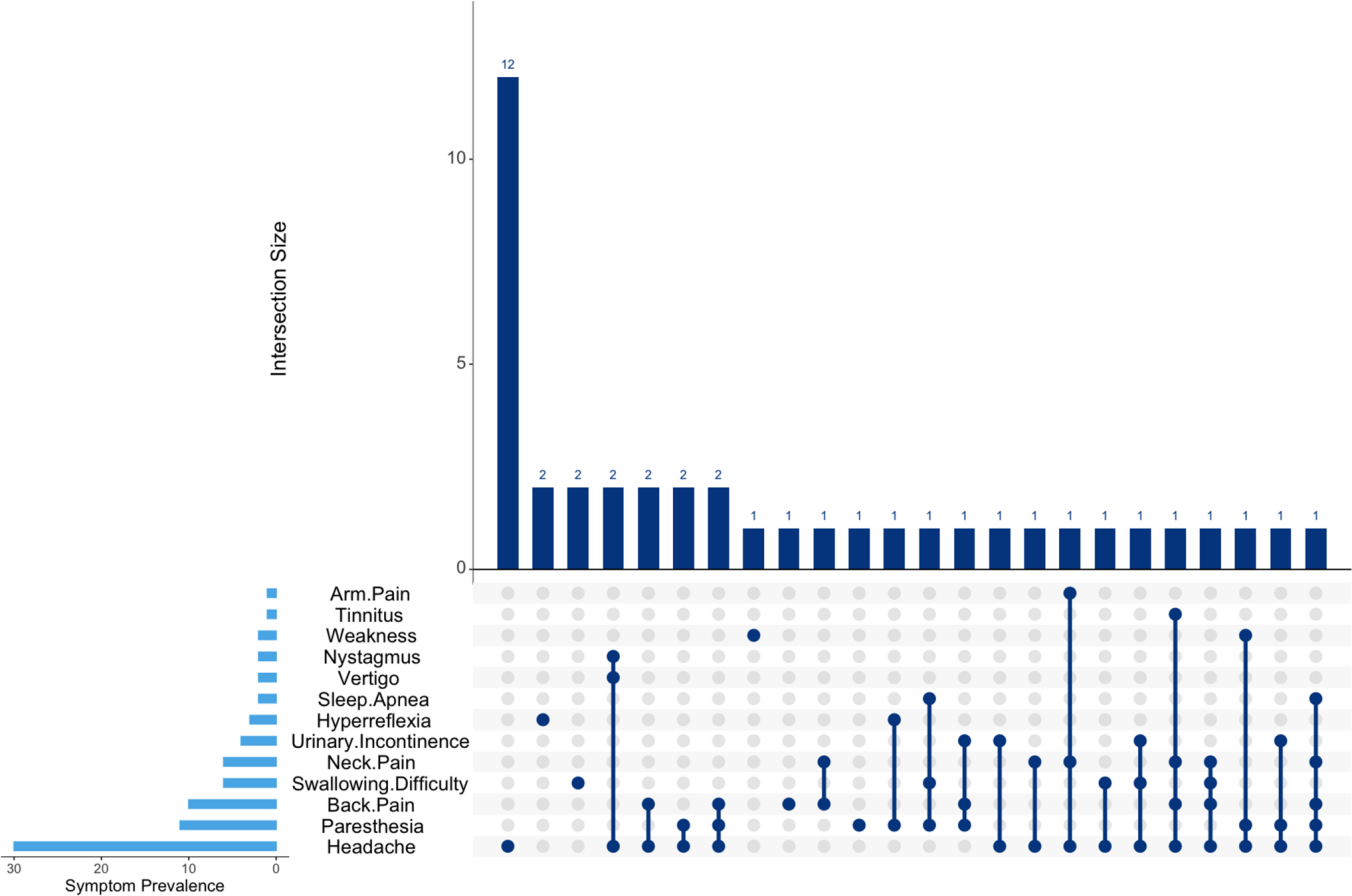
Co-occurrence of preoperative symptoms. An UpSet plot depicting the co-occurrence patterns of preoperative symptoms. The bottom left bar chart indicates the overall prevalence of each symptom, while the top bar chart and matrix show the frequency of various symptom combinations

All patients were symptomatic at the time of surgery. The most frequently reported symptom was headache, present in 73.2% of patients (*n* = 30). Sensory disturbances were also common: paraesthesia occurred in 26.8% (*n* = 11), followed by back pain in 24.4% (*n* = 10), and neck pain in 14.6% (*n* = 6). Arm pain was present in 2.4% (*n* = 1). Brainstem-related symptoms were swallowing difficulty occurred in 14.6% (*n* = 6) and sleep apnea requiring BiPAP in 4.9% (*n* = 2). Urinary incontinence was present in 9.8% (*n* = 4). Vestibular and visual symptoms, such as nystagmus and vertigo, were present in 4.9% (*n* = 2) and 4.9% (*n* = 2) of patients, respectively, while tinnitus was observed in 2.4% (*n* = 1) of patients. Neurological signs included motor weakness in 4.9% of patients (*n* = 2) and hyperreflexia in 7.3% (*n* = 3). Thirteen patients (31.7%) had flexion-extension XR, and none showed instability. Figure 2 illustrates the prevalence of symptoms and demonstrates the co-occurrence of different symptoms.

### Surgical intervention

Surgery was performed by two surgeons (ZT, DT). The most frequent reasons for intervention included persistent symptoms such as headaches, paraesthesia, or brainstem compression accompanied by imaging changes. Surgery was also considered in patients exhibiting syringobulbia or syringomyelia, even if asymptomatic, particularly if these conditions showed an increase in size on surveillance imaging.

All patients underwent foramen magnum decompression (FMD). Preoperative MRI and CT were reviewed for each patient to determine the position of the torcula and relevant vascular anatomy prior to planning the craniectomy. Bony decompression was primarily lateral rather than craniocaudal in extent. The posterior lip of the foramen magnum was routinely removed, along with the posterior arch of C1, with the extent of C1 arch removal guided by the position of the vertebral arteries as identified on preoperative imaging.

The decision between duraplasty and bone-only decompression was individualized. In children younger than five years of age without preoperative syringomyelia, bone-only decompression with dural splitting was performed, as the dura in this age group is often highly vascular and duraplasty carries increased hemorrhagic risk. In older children or those with a preoperative syrinx, the dura was opened and the CSF outflow from the foramen of Magendie was directly inspected. Arachnoid webs around the obex were divided where identified. Duraplasty using Dura-Guard (Baxter) was then performed. Intraoperative ultrasound was used in all cases following bone removal to assess the degree of compression at the craniovertebral junction and to evaluate the adequacy of bony decompression before any decision regarding dural opening was made. While intraoperative ultrasound provided useful qualitative guidance in this series, we acknowledge that robust quantitative parameters defining adequate decompression on ultrasound remain to be established in the literature.

Overall, duraplasty was performed in 32 cases (78.1%); the remainder underwent bone-only decompression with dural splitting.

The median age at surgery was 12 years (IQR 6–14). No major intraoperative complications were recorded. Postoperatively, one patient developed hydrocephalus and pseudomeningocele requiring shunt insertion. Another patient experienced a transient CSF leak that resolved without surgical intervention. The mean follow-up after surgery was 2.1 years (SD ± 1.1). Reoperation was required in 1 case (2.4%) where a second foramen magnum decompression was done because of recurrence of symptoms and a postoperative scan showing insufficient decompression of the posterior fossa structures. A second FMD was performed with an increase in duraplasty.

### Postoperative radiological outcomes

**Fig. 3:**
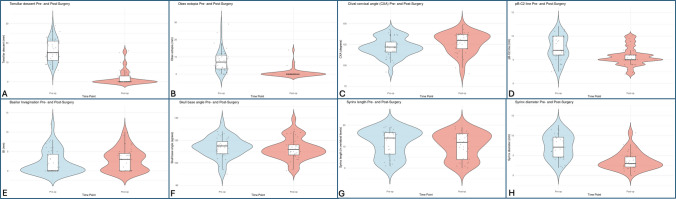
**A–H** Postoperative radiological improvements in tonsillar descent, obex ectopia, CXA, and pB–C2 line, BI, skull base angle, syrinx length, and syrinx diameter. Violin plots illustrating the changes in **A** tonsillar descent (mm), **B** Obex ectopia (mm), **C** clival-cervical angle (CXA, degrees), **D** pB–C2 line (mm), **E** basilar invagination (BI, mm), **F** Skull base angle (degrees), **G** syrinx length (vertebral levels), and **H** syrinx transverse diameter (mm) from preoperative to postoperative states. Statistical significance for these changes is noted in the main text

Postoperative MRI imaging was available for all 41 patients at least six months following surgery. The median tonsillar descent decreased from 15.0 mm (IQR 11.0–21.0 mm) preoperatively to 0.0 mm (IQR 0.0–3.0 mm) postoperatively (*W* = 858.0, *p* < 0.001) (Fig. 3A). Likewise, obex ectopia decreased from a median of 7.0 mm (IQR 3.0–11.0 mm) before surgery to 0.0 mm (IQR 0.0–0.0 mm) after (W = 649.5, *p* < 0.001) (Fig. 3B). Anterior CSF effacement decreased from 80.5% (*n* = 33) to 36.6% (*n* = 15) (*p* < 0.001). Posterior CSF effacement was reduced from 90.2% (*n* = 37) to 41.5% (*n* = 17) (*p* < 0.001).

The CXA angle improved from a median of 122.0° (IQR 116.0–128.0°) to 130.0° (IQR 120.0–137.0°) (*W* = 222.0, *p* = 0.007), indicating reduced medullary kinking (Fig. 3C). Retroflexion of the odontoid (pb–C2 line) decreased significantly from a median of 7.0 mm (IQR 6.0–10.0 mm) to 5.0 mm (IQR 5.0–6.0 mm) (*W* = 563.5, *p* < 0.001) (Fig. 3D).

Since 33 of 41 patients had no BI preoperatively, the overall median was 0.0 mm (IQR 0.0–4.3 mm); postoperatively, the median rose to 3.0 mm (IQR 0–4.5 mm). However, this change did not reach significance across the entire cohort (*W* = 67.0, *p* = 0.074) (Fig. 3E). The BAI decreased from 7.0 mm (SD ± 2.8) to 6.7 mm (SD ± 2.2) (*t*(40) = 0.6, *p* = 0.50), and the BDI decreased from 5.7 mm (SD ± 2.1) to 5.2 mm (SD ± 1.7) (*t*(40) = 1.3, *p* = 0.18).

Changes in the skull base angle did not reach significance, with a preoperative median of 115.0° (IQR 109.0–119.0°) versus 112.0° (IQR 107.0–116.0°) after surgery (*t*(40) = 1.1, *p* = 0.28) (Fig. 3F).

Preoperative syringobulbia was present in 8 patients (19.5%) and persisted after surgery in only 1 (2.4%). Among the 31 patients with preoperative syringomyelia, 27 improved (87.1%) and 4 remained unchanged (12.9%). Of these 4, 3 had persistent posterior effacement, and 2 had persistent anterior effacement postoperatively, while 1 had neither. The maximum transverse syrinx diameter fell from a median of 7.1 mm (IQR 4.5–9.7 mm) to 3.0 mm (IQR 2.0–4.7 mm) (*W* = 413.0, *p* < 0.001) (Fig. 3G). Syrinx length similarly decreased from a median of 14 vertebral levels (IQR 6.0–17.0) to 12 levels (IQR 4.0–16.0) (*W* = 190.5, *p* = 0.001) (Fig. 3H). Overall, all parameters showed significant improvement except skull base angle and BI distance.

### Postoperative symptom outcomes

**Fig. 4:**
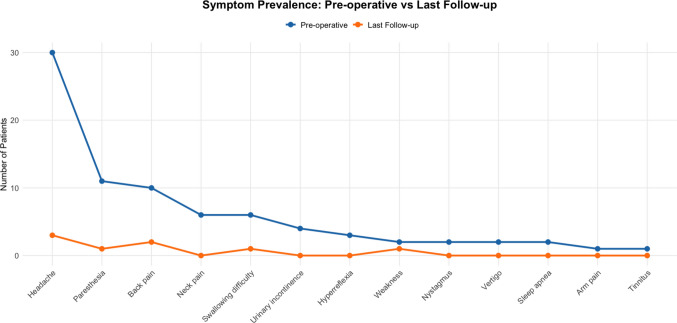
Symptom prevalence before surgery and at last follow-up. Line graph showing the number of patients reporting each symptom at the preoperative baseline and at the last follow-up (mean follow-up: 25.1 months). A marked reduction in symptom burden was observed across most domains, with several symptoms resolving completely by the final assessment

Symptoms were assessed at presentation and at last follow-up (mean 25.1 months; SD ± 12.8). Figure 4 shows the progression of the symptoms.

Headache was the most prevalent preoperative symptom, reported by 30 patients (73.2%), and decreased markedly to 3 patients (7.3%) at last follow-up (*p* < 0.001). Paraesthesia, initially present in 11 patients (26.8%), resolved in all but one patient (2.4%) by final follow-up (*p* = 0.003). Back pain affected 10 patients (24.4%) before surgery and was present in 2 patients (4.9%) at last follow-up (*p* = 0.004). Swallowing difficulty, noted in 6 patients (14.6%) preoperatively, was reported by 1 patient (2.4%) at final follow-up (*p* = 0.062). Weakness, which affected 2 patients (4.9%) before surgery, persisted in 1 patient (2.4%) at the last follow-up (*p* = 1.000).

Other symptoms that resolved completely by last follow-up included neck pain (14.6 to 0%; *p* = 0.031), urinary incontinence (9.8 to 0%; *p* = 0.12), hyperreflexia (7.3 to 0%; *p* = 0.25), nystagmus (4.9 to 0%; *p* = 0.50), vertigo (4.9 to 0%; *p* = 0.5), arm pain (2.4 to 0%; *p* = 1.00), sleep apnea (4.9 to 0%; *p* = 0.50) and tinnitus (2.4 to 0%; *p* = 1.00). Table 1 summarizes the results of the statistical tests.

**Table 1:** Symptoms evolution with statistical analysis

Symptom	Pre-operative (*n*, %)	Last Follow-up (*n*, %)	McNemar’s *χ*^2^ (1, *N* = 41)	*p*-value
Headache	30 (73.2%)	3 (7.3%)	27	< 0.001
Paraesthesia	11 (26.8%)	1 (2.4%)	8.3	0.003
Back pain	10 (24.4%)	2 (4.9%)	8	0.004
Neck pain	6 (14.6%)	0 (0%)	6	0.031
Swallowing difficulty	6 (14.6%)	1 (2.4%)	5	0.062
Urinary incontinence	4 (9.8%)	0 (0%)	4	0.125
Hyperreflexia	3 (7.3%)	0 (0%)	3	0.25
Weakness	2 (4.9%)	1 (2.4%)	0.3	1
Nystagmus	2 (4.9%)	0 (0%)	2	0.5
Vertigo	2 (4.9%)	0 (0%)	2	0.5
Sleep Apnea	2 (4.9%)	0 (0%)	2	0.5
Arm pain	1 (2.4%)	0 (0%)	1	1
Tinnitus	1 (2.4%)	0 (0%)	1	1

## Discussion

**Fig. 5:**
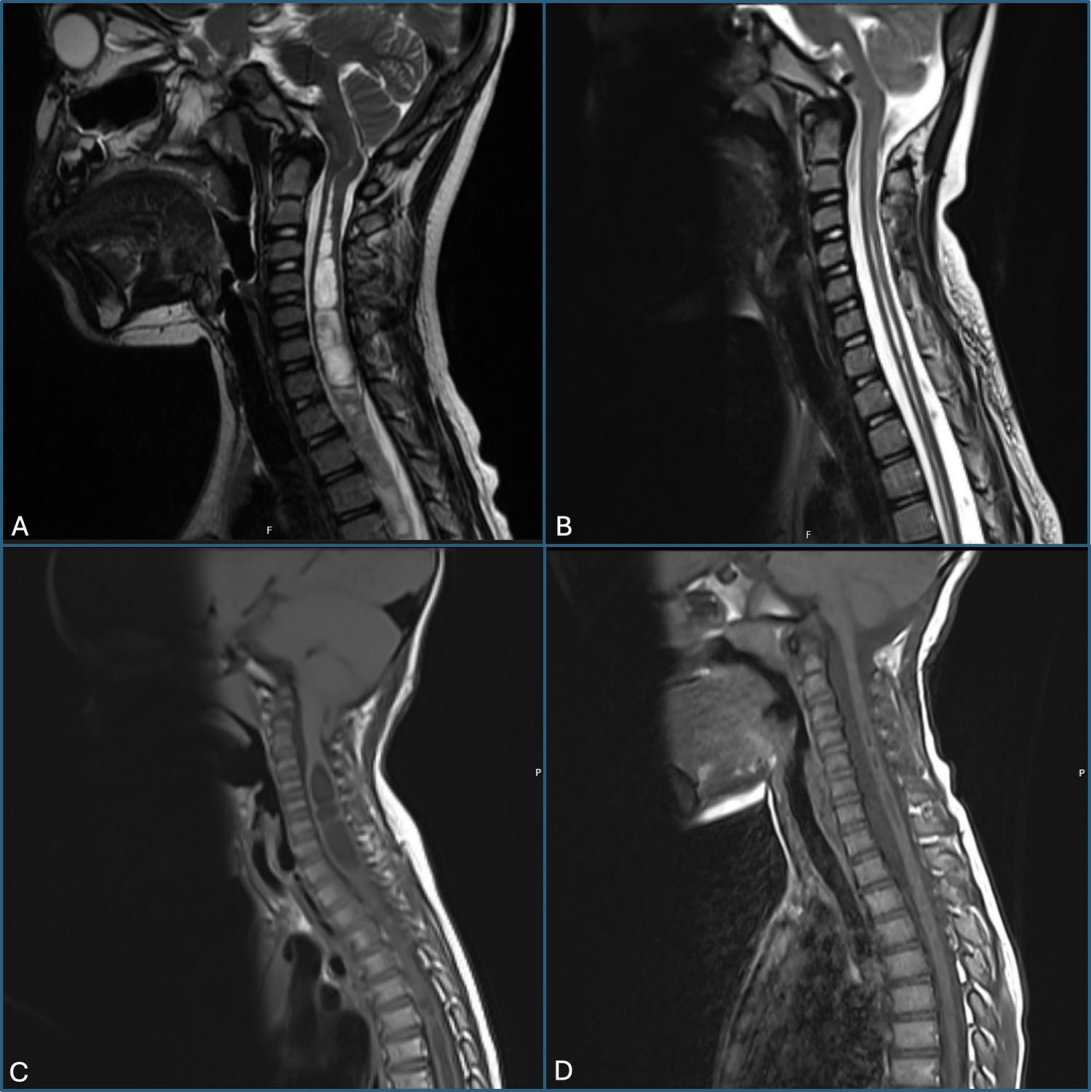
Decompression in posterior pathology. Illustrative sagittal T1-weighted MRI images demonstrating effective posterior fossa decompression and realignment of neural structures and improvement of syringomyelia. **A** Preoperative scan showing significant tonsillar descent and brainstem crowding. **B** Postoperative scan demonstrating reduced tonsillar descent and restoration of cerebrospinal fluid flow. **C** Preoperative scan showing significant tonsillar descent, brainstem crowding, and cervical syringomyelia. **D** Postoperative scan demonstrating reduced tonsillar descent and restoration of cerebrospinal fluid flow with significant reduction in syringomyelia length and width

In this study, we investigated the outcomes of FMD in a pediatric cohort with CCM, characterized by tonsillar descent greater than 5 mm in combination with at least one other craniovertebral junction (CVJ) abnormality—70.7% exhibiting craniocervical deformity, 36.6% showing retroflexed odontoid, 24.4% platybasia, and 19.5% diagnosed with basilar invagination. All patients were symptomatic, with headaches (73.2%) being the most frequent complaint. FMD alone produced clinical and radiological improvements. These improvements are comparable to those reported for “simple CM”. Notably, significant reductions were observed in tonsillar descent (median 15.0 to 0.0 mm) and obex ectopia (median 8.0 to 0.0 mm), accompanied by restoration of CSF flow and marked symptomatic relief (Fig. 5A, B). Specifically, headache prevalence dramatically decreased to just 7.3% at the last follow-up. Syringomyelia and syringobulbia also significantly regressed in most cases, with median syrinx diameter reducing from 7.0 to 3.0 mm and length from 14 to 12 vertebral levels (Fig. 5C, D). These outcomes underscore that posterior fossa decompression, even in anatomically challenging cases, can effectively realign neural structures and restore physiological CSF dynamics without the need for additional procedures.

Persistent CSF space effacement following FMD warrants consideration, as more than a third of patients in this cohort had residual anterior or posterior effacement on postoperative imaging. Despite this, the clinical significance of persistent effacement appeared limited: most of these patients were symptom-free at last follow-up, with only 2 reporting ongoing symptoms. A partial association was observed between persistent effacement and failure of syrinx improvement; 3 of 4 patients with unchanged syrinx had residual posterior effacement, and 2 had residual anterior effacement, suggesting that incomplete restoration of CSF spaces may be a marker for suboptimal syrinx response rather than a direct driver of clinical symptoms.

Nevertheless, none of the patients had symptoms of instability and required occipitocervical (OC) fixation post-FMD during the follow-up period, including those with craniocervical deformity or retroflexed odontoid and morphometric values previously associated with high fixation risk. While the pB–C2 distance and CXA did show modest postoperative improvement, the degree of change was modest. Nevertheless, in individual cases, these shifts correlated with reduced brainstem compression, suggesting that decompression may provide biomechanical benefits even in anterior pathology.

It has been suggested that FMD alone might exacerbate craniocervical deformity; however, in this series, basilar invagination (BI) and skull base angle measurements showed no statistically significant differences before and after surgery.

Our follow-up extends until patients transfer to the adult unit, during which time occipitocervical (OC) fixation is considered only in the presence of craniocervical instability, characterized by excessive occipitocervical or C1–C2 motion. In cases of persistent or recurrent symptoms after FMD, a repeat MRI is obtained to assess CSF flow across the craniocervical junction. If inadequate decompression or restricted CSF flow is identified, a re-do FMD is indicated rather than OC fixation. However, if postoperative imaging confirms adequate decompression with restored CSF flow and symptoms persist, particularly in association with radiological evidence of ventral brainstem compression or instability, OC fixation is then considered. Notably, within our mean 2-year follow-up period, no patient developed craniocervical instability or required OC fixation.

This study is small, and follow-up is limited to 25.1 months; however, the findings question the notion that CM occurring with complex craniocervical junction abnormalities invariably requires concurrent or staged occipitocervical fusion. Historically, concepts such as Complex Chiari I Malformation (CCM), initially described by Grabb et al. [5] for Chiari I with retroflexed odontoid, and Chiari 1.5, defined by Tubbs et al. [22] as including obex ectopia, have frequently implied an increased requirement for OC fixation, particularly when associated with syrinx. Bollo et al. [3] further defined factors such as obex ectopia, a pB-C2 distance > 9 mm, a CXA < 125°, or any type of BI as high-risk indicators for fusion. Collectively, these reports suggest that significant symptom resolution is difficult to achieve without OC fixation. The significance of these findings has, however, been questioned by other authors [23], and our experience also suggests that FMD alone may be sufficient to deal with the Chiari symptoms and syringomyelia in these patients.

A closer comparison with the literature, particularly the work of Bollo et al. [3], reveals important distinctions regarding surgical necessity. Their cohort, despite exhibiting comparable mean morphometric values for pB-C2 distance (10.2 mm) and CXA (115.5°), ultimately required OC fixation. In contrast, our patients presenting with medullary kinking or retroflexed odontoid, who had similar mean pB-C2 distances (10.1 mm) and CXA (117.2°), did not subsequently require OC fixation. These findings reinforce our conservative approach to OC fixation, which prioritizes clinical progression and post-FMD imaging, thereby potentially reducing surgical burden without compromising efficacy.

While acknowledging that BI with severe brainstem compression is a strong indication for OC fixation, as documented by Goel et al. [11], our cohort’s characteristics differed. Although BI was present in 8 patients, the median odontoid process tip above McGregor’s line was 6.5 mm (IQR 6–7.5), suggesting less severe displacement. Moreover, none of our patients exhibited cranial nerve palsies or respiratory compromise, symptoms commonly associated with severe anterior compression requiring fixation. These mitigating factors likely contributed to the successful management of BI with FMD alone in our series.

This study contributes to a growing body of evidence supporting FMD as a standalone treatment for CCM. The debate around the necessity of routine fusion in this population has been critically examined by others. Wagner et al. [24] conducted a comprehensive review challenging the hypothesis that atlantoaxial instability is the universal progenitor of CVJ abnormalities, including Chiari I and basilar invagination, concluding that current evidence is insufficient to justify abandoning established surgical strategies, including posterior fossa decompression, in favor of a single universal fusion approach. Similarly, Deora et al. [25] concluded that in patients with pure Chiari I Malformation without atlantoaxial dislocation or basilar invagination and with symmetrical C1–2 joints, posterior fossa decompression alone achieves neurological improvement in over 70% of cases, and that routine cervical stabilization constitutes an overkill in this subset. Our findings align with and extend these conclusions to the CCM population specifically, including patients with retroflexed odontoid, medullary kinking, and basilar invagination. Furthermore, our findings align with those of Giallongo et al. [26] and Kotil et al. [27], although our study provides more robust radiological data and includes patients with a broader range of CVJ anomalies. Furthermore, our detailed morphometric analysis demonstrates that decompression not only alleviates crowding but also facilitates subtle yet meaningful realignment of the brainstem. The absence of major surgical complications and the low reoperation rate further highlight the safety and durability of this approach. One patient required a second FMD due to insufficient initial bone removal, which resolved the recurrent symptoms, reinforcing the importance of intraoperative assessment and postoperative imaging in guiding surgical adequacy.

However, we must acknowledge the limitations of our study. This was a retrospective, single-center study with a median follow-up of two years. Longer-term monitoring is essential, particularly in patients with syndromic features or borderline instability. Additionally, while clinical improvement was evident, standardized neuropsychological assessments and quality-of-life measures would offer deeper insight into patient outcomes.

In conclusion, our findings question the notion that CCM requires fusion by demonstrating that FMD alone can restore function and alleviate symptoms in most pediatric CCM patients. We suggest that patients with CM and suspicion of instability should get preoperative dynamic X-rays to confirm the presence of excessive movement. This is the cohort where OC fixation should be considered along with decompression. This paradigm shift may spare children from the morbidity of unnecessary fixation and encourage further investigation into the functional understanding of Chiari-related pathology.

## Conclusion

This study suggests that foramen magnum decompression (FMD) alone is an effective and safe surgical strategy for pediatric patients with features of complex Chiari I Malformation (CCM). FMD successfully achieved substantial clinical and radiological improvements, including resolution of symptoms, regression of syringomyelia/syringobulbia, and demonstrable anatomical realignment without the requirement for instrumentation.

## Data Availability

No datasets were generated or analysed during the current study.
